# Aberrant Water Structure Dynamics in B16 Melanoma-Bearing Mice by Time Domain Refractometry Analysis

**DOI:** 10.3390/biology12091250

**Published:** 2023-09-18

**Authors:** Kahori Furuhata, Haruchika Masuda, Atsuko Sato, Kumiko Miyata, Naoki Shinyashiki, Rio Kita, Kotaro Imagawa, Tadashi Akamatsu, Shin Yagihara

**Affiliations:** 1 Department of Physiology, School of Medicine, Isehara Campus, Tokai University, 143 Shimokasuya, Isehara 259-1193, Japan; frht.khr@gmail.com (K.F.); aasatoaug@gmail.com (A.S.); m-kumiko@ymail.ne.jp (K.M.); 2 Regenerative Medicine Research Division, Shonan Research Institute of Innovative Medicine, Shonan Kamakura General Hospital, 1370-1 Okamoto, Kamakura 247-8533, Japan; 3 Department of Plastic Surgery, School of Medicine, Isehara Campus, Tokai University, 143 Shimokasuya, Isehara 259-1193, Japan; imagawa@is.icc.u-tokai.ac.jp (K.I.); akamatu@is.icc.u-tokai.ac.jp (T.A.); 4 Department of Nutritional Science, Faculty of Applied Biosciences, Setagaya Campus, Tokyo University of Agriculture, 1-1-1 Sakuragaoka, Tokyo 156-8502, Japan; 5 Department of Physics, School of Science, Shonan Campus, Tokai University, 4-1-1 Kitakaname, Hiratsuka 259-1292, Japan; naoki-ko@keyaki.cc.u-tokai.ac.jp (N.S.); rkita@keyaki.cc.u-tokai.ac.jp (R.K.); 8gihara.wsal@gmail.com (S.Y.); 6 Micro/Nano Technology Center, Shonan Campus, Tokai University, 4-1-1 Kitakaname, Hiratsuka 259-1292, Japan

**Keywords:** water structure dynamics, time domain reflectometry, tumor

## Abstract

**Simple Summary:**

Living bodies comprise approximately 55–75% water to maintain homeostasis. However, little is known about the differences in in vivo water molecule dynamics (water structure dynamics; WSD) under physiological and pathophysiological statuses. Here, we examined the WSD of ex vivo samples in tumor-bearing mice versus healthy mice by dielectric spectroscopy using time domain reflectometry. Tumor tissues, from early after engrafting, exhibited remarkable features of WSD close to pure water, different from the other organs of tumor-bearing and healthy mice. Further, certain organs in tumor-bearing mice temporally revealed different WSD features compared to those in healthy mice. Conclusively, tumor-bearing mice revealed aberrant WSD, unlike healthy mice. Thus, dielectric spectroscopy in terms of WSD may provide novel pathophysiological perspectives in living organisms.

**Abstract:**

Living bodies comprise approximately 55–75% water to maintain homeostasis. However, little is known about the comprehensive differences in in vivo water molecule dynamics (water structure dynamics; WSD) between physiological and pathophysiological statuses. Here, we examined the WSD of ex vivo tumor tissues and organs from tumor-bearing mice with engrafted mouse malignant melanoma cells (B16-F10) in the right flanks to compare with those in healthy mice, using time domain reflectometry of dielectric spectroscopy at days 9, 11, and 14 after engrafting. The relaxation parameters of relaxation time (*τ*), relaxation time distribution parameter (*β*), and relaxation strength (∆*ε*) were measured on tumor tissues and lung, liver, kidney, and skin tissues. Immediately afterward, the water contents (%) in the tumor and the other organs were calculated by measuring their weights before and after freeze-drying. Each parameter of the tumor was compared to that of pooled values of other organs in tumor-bearing (TO) and healthy mice (HO). The tumor water content temporarily increased compared to that of HO at day 11; the tumor volume was also prone to increase. In contrast, tumor tissues exhibited significantly higher values of *β* close to 1 of ultrapure water and ∆*ε* compared to TO and HO at all times. Moreover, *β* in the viscera of TO was prone to increase compared to that of HO with significantly higher levels at day 11. Conclusively, tumor-bearing mice exhibited systemically aberrant WSD, unlike healthy mice. Thus, dielectric spectroscopy in terms of WSD may provide novel pathophysiological perspectives in tumor-bearing living bodies.

## 1. Introduction

The water content in humans and mice amounts to approximately 55% to 75% [1,2]. In vivo, water plays an essential role in homeostatic maintenance, as living organisms are promised survival with a well-controlled internal water content responding to in vivo environments [1]. The macroscopic water dynamics in pathological aspects, including edema, dehydration, and pleural and ascitic fluid, influence the microscopic behavior of in vivo water molecules.

In general, the behavior of in vivo water molecules, i.e., water structure dynamics (WSD) is classified as ‘restraint or bound water’ or ‘free water’ with or without movement restricted by particularly high polymer organic matter [3,4,5,6]. Microscopic WSD in living bodies is assumed to dynamically transition to maintain homeostasis in response to diverse environments.

Dielectric relaxation measurement for water molecules is an analytical method to characterize WSD from the frequency dependence of the complex dielectric constant obtained by the rotational diffusion motion of the permanent dipole moment of the water molecules in an electric field [7,8,9].

In a specific frequency range, the dielectric constant is obtained according to the water content. For biological samples, the molecular behaviors of nucleic acids, biological cells, tissues, proteins, and water are observed in order from the low-frequency range [10,11,12,13]. The measurement technique is simple and rapid, and the use of coaxial open-ended electrodes enables non-invasive and non-destructive measurement of biological tissues. Therefore, the dielectric constant has been used to measure various biological tissues and cells.

Previous studies have performed dielectric spectroscopy measurements to determine the differences in WSD between intact and pathological samples. For instance, in the tissues resected from patients with colon [14,15] and liver [16] cancers, or brain tumors [17], dielectric measurements exhibit a unique pattern with higher dielectric constants compared to intact samples at similar frequencies. The differences in dielectric polarization between injured and non-injured sciatic nerves in the rat nerve crush injury model have been revealed, which might be caused by inflammation and edema [18]. Thus, researchers have sought to differentiate lesional tissues from intact ones using dielectric measurements as a novel diagnostic tool. Dielectric spectroscopy revealed that WSD is affected by various molecules, including organic polymers or mineral ions in the living body [10,12,13]. Considering that those in vivo molecules or mineral ions dynamically sway between pathological and physiological environments, the reported feature of in vivo WSD is readily inferred. We have been discussing dynamics of water molecules per se as ‘WSD’, using dielectric relaxation parameters obtained for a high frequency process usually observed at the dielectric high frequency range at around 10 GHz, where the dielectric relaxation effect of organic polymers and ions could be excluded [13]. Therefore, in the high frequency region, relaxation parameter analysis enables us to capture the extent of spatial hydrogen bond network spread and fragmentation of water molecules as WSD. Herein, we tried to investigate whether ‘WSD’ in tumor tissue and other organs (TO) in tumor-bearing mice may differ from that in organs in healthy mice (HO) in the high frequency region, using a dielectric relaxation measurement method: time domain reflectometry (TDR).

## 2. Materials and Methods

### 2.1. Construction of B16 Tumor-Bearing Mice

The animal experiment was performed with the approval of the Animal Experimental Committee (Reference No. 212024) of Tokai University School of Medicine. Mouse malignant melanoma cells (B16-F10) were cultured with 10% FBS-DMEM (High Glucose) (FUJIFILM Wako Pure Chemical Co. Ltd., Osaka, Japan) medium added to L-glutamine and penicillin-streptomycin in a 25 cm^2^ flask (BM Equipment Co. Ltd., Tokyo, Japan) at 37 °C in 5% CO_2_ incubator. B16-F10 cells cultured with 80% confluency were harvested with 0.05% trypsin-EDTA (Thermo Fisher Scientific Co. Ltd., Waltham, MA, USA) and suspended at 5 × 10^5^ cells/100 μL of DMEM alone after washing with PBS. Cell suspension (100 µL) was injected into the right flank of 8-week-old male C57BL/6J mice (The Jackson Laboratory Japan Inc., Yokohama, Japan), and an equal volume of DMEM was injected into the corresponding site of healthy mice.

### 2.2. Dielectric Relaxation Measurement

**Figure 1:**
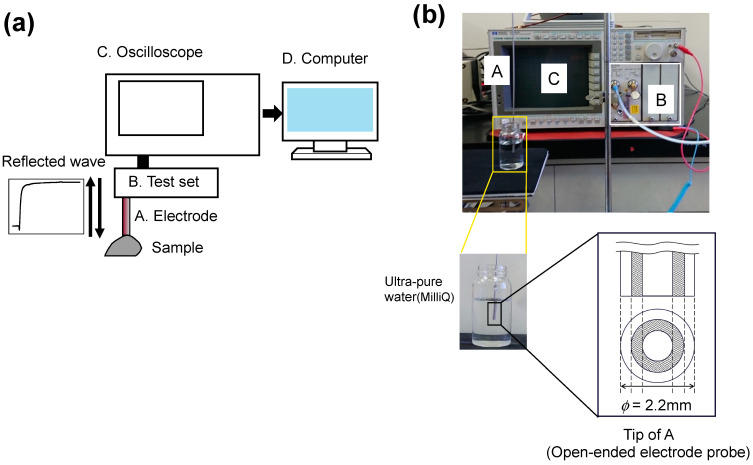

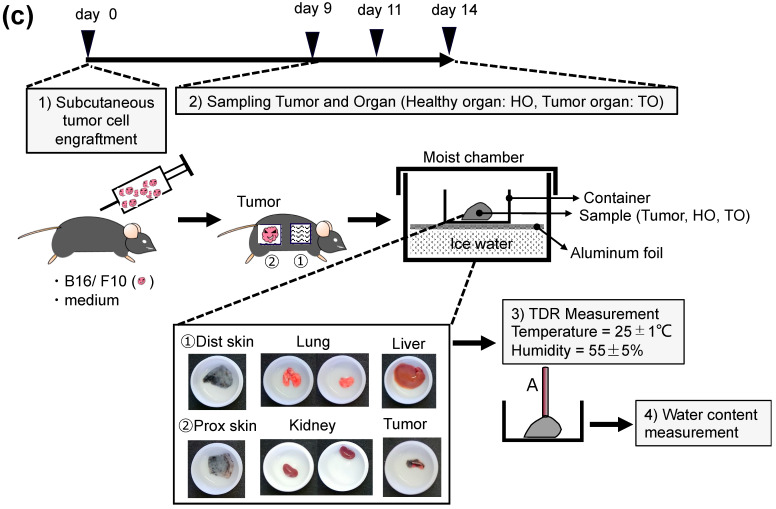
The experimental protocol using time domain refractometry (TDR). The scheme (**a**) and image (**b**) of TDR system. The probe tip of an open-ended electrode with a 2.2 mm diameter shown in (**b**). (**c**) The experimental protocol showing (1) to (4) procedures. HO: organs in healthy mice; TO: organs in B16 melanoma tumor-bearing mice. Five mice in each group were subjected to the experiment. The average value, following each measurement of the right and left organs in the lung and kidney, was used for statistical analysis.

As previously reported [3], an open-ended coaxial electrode with a 2.2 mm outside diameter and a 30 cm length (COAX Co. Ltd., Yokohama, Japan) was connected to a 2 m flexible cable connected to a test set (HP54753A; Hewlett-Packard Inc., Palo Alto, CA, USA) with an oscilloscope (HP54750; Hewlett-Packard Inc.). A coaxial open-ended electrode with a *γd* 0.172 mm electric length was covered with an external conductor of stainless steel to prevent electrode corrosion. Acetone (special grade, Fujifilm Wako Pure Chemicals Co. Ltd., Osaka, Japan) and dimethyl sulfoxide (special grade, Fujifilm Wako Pure Chemicals Co. Ltd., Osaka, Japan) were used as references. The measurement frequency range was 10 MHz–30 GHz. The sample dielectric constant was measured by contacting the tip of coaxial open-ended electrode under the conditions of 25 ± 1 °C and humidity 55 ± 5% (Figure 1a,b).

### 2.3. Sampling of the Organ and Tumor Tissues for TDR

At each time point on days 9, 11, and 14 after tumor cell injection, tumor sizes were measured (the width (W) and longitudinal length (L)) with a vernier caliper, and tumor volumes were calculated with the formula W^2^ × L/2 mm^3^. After that, mice underwent intraperitoneal injection of pentobarbital sodium (150 mg/kg) (Somnopentyl; Kyouritsu Pharmaceutical Co. Ltd., Tokyo, Japan) for euthanization. Immediately after anesthetization, blood was aspirated with a heparinized tuberculin syringe by heart puncture. Subsequently, the tumor and other organs (lung, liver, kidney, skin) were resected in tumor-bearing and healthy mice. The skin close to the tumor at the right flank was defined as proximal skin (prox skin), and the distant skin at the right shoulder (dist skin) in tumor-bearing mice was at 1 cm^2^ (W × L = 1 cm × 1 cm). Each ectopic skin in the tumor-bearing mice was excised in healthy mice. The right and left whole lungs or kidneys and the left lobes of the liver were resected for the dielectric measurement. Each sample of tumor tissues and the other organs put in a plastic container (Cat No. 01752; Sanplatec Co. Ltd., Osaka, Japan) was placed on aluminum foil on top of ice in a moisture chamber until measurement to avoid freezing and drying (Figure 1c).

### 2.4. Analytical Method of the Dielectric Relaxation Result of a Measurement

When an AC electric field is applied to a sample, the electric dipole moments of the constituent molecules are oriented and polarized. The behavior of the molecules can be characterized by the frequency dependence of the complex dielectric constant ($\varepsilon^{*}$). The obtained complex dielectric constant can be expressed as an equation [19]:

(1) $$\varepsilon^{*}=\varepsilon^{\prime }-j\varepsilon^{\prime \prime }$$

In the formula, $\varepsilon^{\prime }$ is called the real part and indicates the charge bias of the dielectric constant, and $\varepsilon^{\prime \prime }$ is the imaginary part and represents the energy loss due to the collision of molecules. *j* is an imaginary unit represented by *j*^2^ = −1. In this study, TDR was used for dielectric measurements [20,21,22]. In TDR, a fast-rising step pulse wave with a rise time of 35 ps is injected into the sample, and the reflected pulse is observed. The analysis of the reflection pulse by the Laplace Fourier transform allows the detection of frequency dependence of the complex dielectric constant [19]. The obtained complex dielectric constant can be expressed as a dielectric relaxation curve by noting the complex dielectric constant on the y-axis as $\varepsilon^{\prime }$ or $\varepsilon^{\prime \prime }$ and the frequency on the x-axis. The difference method in TDR measurement takes the difference between the reflected pulses from the unknown sample and the known sample of the dielectric constant and analyzes the waveform.

In the dielectric relaxation measurement, curve-fitting analysis using the relaxation function was used to determine the relaxation parameters (relaxation time: τ, relaxation strength: ∆ε, and relaxation time distribution parameter: β) to discuss the molecular behavior in detail. The following equation, called Debye’s equation [23], can successfully describe the experimental values of water (Figure S1):

(2) $$\begin{array}{c} \varepsilon^{*}=\varepsilon_{\infty }+\frac{∆\varepsilon }{1+j\omega \tau } \end{array}$$

$$∆\varepsilon =\varepsilon_{S}-\varepsilon_{\infty }$$

Here, $\varepsilon_{\infty }$ is the lowest dielectric constant in the high-frequency limit, $\varepsilon_{S}$ is the highest dielectric constant in the low-frequency limit for a relaxation process, ω is the angular frequency, and τ is the time required for molecular orientation. The frequency (f) is obtained from the equation τ=1/2πf. The inflection of the graph curve of the relation of $\varepsilon^{\prime }$ and log_10_ f provides the maximum peak frequency ($1/2\pi f_{max}$) in the graph curve of $\varepsilon^{\prime \prime }$ and log_10_ f in Figure S1. Here, the relaxation intensity ∆ε correlates with the water content. In aqueous polymer solutions and gels, Debye’s equation cannot be used in many cases. Therefore, Cole introduced a contrasting β to derive the following empirical equation [24,25] called the Cole–Cole equation (Figure S2):

(3) $$\varepsilon^{*}=\varepsilon_{\infty }+\frac{∆\varepsilon }{1+{\left(j\omega \tau \right)}^{\beta }},\;\left(0<\beta \leq 1\right)$$

When detecting 1 of the β value as a homogeneous water molecule motion, anything smaller than that can be considered a non-uniform water molecule motion. Therefore, if the pure water β is taken as the reference, the β can be regarded as the uniformity of water molecule motion in direct electrical conduction. In this experiment, the curve-fitting analysis was performed using the following two Cole–Cole equations and the additional components of direct current (DC) electrical conduction, where the low-frequency and high-frequency processes are denoted by *l* and *h*, respectively (Figure S3):

(4) $$\varepsilon^{*}=\varepsilon_{\infty }+\frac{\Delta \varepsilon_{l}}{1+{\left(j\omega \tau_{l}\right)}^{\beta_{l}}}+\frac{\Delta \varepsilon_{h}}{1+{\left(j\omega \tau_{h}\right)}^{\beta_{h}}}-j\frac{\sigma_{\mathrm{DC}}}{\varepsilon_{0}\omega }$$

$\sigma_{\mathrm{DC}}$ is the DC conductivity determined by $\sigma_{\mathrm{DC}}/\varepsilon_{0}\omega$ in the extreme low-frequency range, where the conductivity originates from ions and macromolecules contained in living organisms. In the present study, curve-fitting procedures were performed in the frequency range of 100 MHz–30 GHz by tentatively deciding relaxation parameters for *l* and *h* processes: in the real part, leading to excluding the effect of lower frequency processes; in the imaginary part, shown as the contribution term of DC electrical conduction from the lower frequency region. The analysis and discussion were carried out in the *h* frequency range, where the relaxation process of water molecules alone is observed.

### 2.5. Measurement of the Water Content

Immediately after dielectric constant measurement, the weight of each sample was measured as wet weight and then frozen at −80 °C followed by freeze-drying. For freeze-drying, the frozen samples were dried with a lyophilizer (VirTis BenchTop K; SP Scientific Co. Ltd., Warminster, PA, USA) for 24 h. Immediately after freeze-drying, the dried weight was measured. The % water content was calculated by subtracting the dry weight from the wet weight and dividing the result by the wet weight.

### 2.6. Statistical Analysis

Two-way analysis of variance with post-hoc Tukey’s multiple comparison tests among the groups during the observation period was performed using Prism 9 software (Graph Pad Software Inc., SanDiego, CA, USA). *p* < 0.05 was evaluated as statistical significance.

## 3. Results

### 3.1. How do Tumor Volume and Mass Vary during the Observation Period?

**Figure 2:**
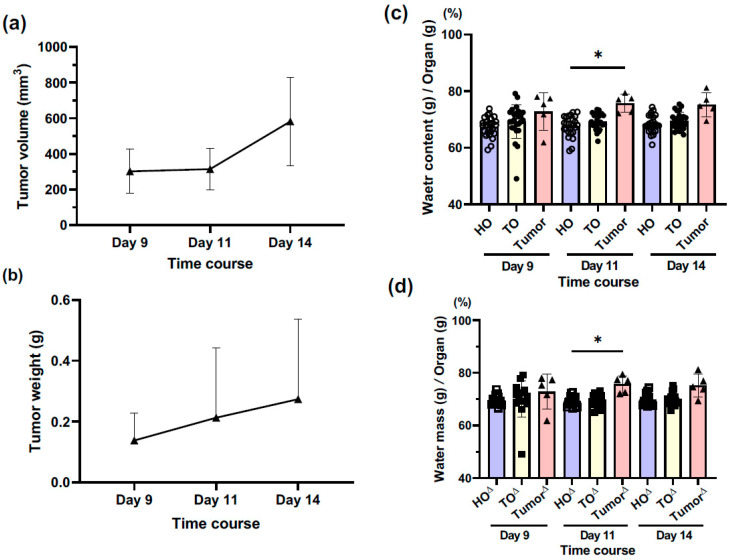
The tumor growth of engrafted B16 melanoma and the water contents of organs and tumors in healthy and tumor-bearing mice. (**a**) Tumor tissue volume (mm^3^) and (**b**) tumor tissue weight (g) calculated using the caliber measurement. The graphs are presented as mean ± SD, n = 5. (**c**,**d**) The comparison of water contents among tumor tissues and whole organs in healthy (HO) and tumor-bearing (TO) mice. The graphs are presented as mean ± SD. (**c**) HO and TO with both skins (n = 25). (**d**) HO^∆^ and TO^∆^ without both skins (n = 15). ^∗^ *p* < 0.05.

After tumor cell transplantation, the tumor size and weight gradually increased over time after B16 melanoma cell transplantation, although not significantly (Figure 2a,b, Table S1).

### 3.2. How Does the Water Content of Tumor Tissues Change over Time?

The water content of tumor tissues tended to increase compared to that of organs of healthy mice (HO) and tumor-bearing mice (TO) over time; a significant difference was found at day 11 (Figure 2c, Table S2). Similar results were obtained in the comparison of tumor tissues and HO and TO, excluding the proximal and distal skins (Figure 2d, Table S2). There was no difference between HO and TO in the lung, liver, kidney, and dist skin, while the prox skin in TO exhibited a significantly higher water content compared to that in HO (Figure S4, Table S6). This result was thought to be because the prox skin is strongly affected by the tumor tissue with hyperpermeability of the tumor vasculature.

### 3.3. How Is It Consistent with Existing Measurements of Dielectric Constant and Conductivity?

The consistency of the measured dielectric constant and conductivity to the online database (https://itis.swiss/virtual-population/tissue-properties/database/tissue-frequency-chart/, accessed on 11 August 2023) was confirmed with comparison of those values at 1 GHz between them, shown in Figure S5. Notably, an outlier is only recognized in the kidney in both the dielectric constant and conductivity. However, the ratios of the online database to the averaged experimental data are 1.4- and 1.8-fold, respectively; their ratios in the other organs are 0.8 to 1.9 in the dielectric constant and conductivity. There are no difference in the ratios between this experiment and the online database that are more than several times. Taken together, the consistency of our mouse data with the online database for the organs seems to approximately remain. Here, the lung and skins tended to exhibit a lower dielectric constant, compared to the viscera of kidney and liver in both of the data sources (Figure S5c). In the air-including lung, compared to the solid viscera, the dielectric constant is considered to inevitably decline, due to the quite low dielectric constant of around 1 of air per se. Furthermore, in the skin, the presence of the epidermal stratum corneum with a low level of water content is assumed to contribute to the reduction in the dielectric constant.

### 3.4. Does Tumor Tissue Exhibit a Different Dielectric Relaxation Curve Than Other Organs?

**Figure 3:**
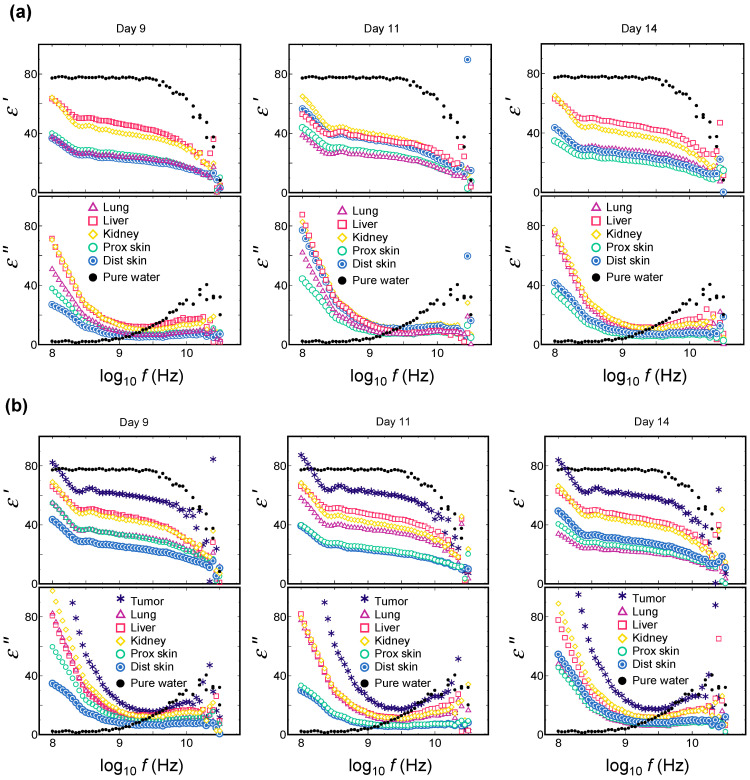
The representative real and imaginary curves of complex dielectric constant: dielectric dispersion and absorption curves at each time point by TDR. (**a**) The organs in healthy mouse. (**b**) The organs and tumor tissues in tumor-bearing mouse.

In HO, the solid viscera, such as the liver and kidney, indicated a higher dielectric constant and dielectric loss (*ε*′ and *ε*″) than the skin and the lung over time. Contrarily, both the skin and the lung exhibited similar behavior at >9 GHz frequency (Figure 3a). In TO, the solid viscera and both skins indicated similar features to HO, while the tumor tissue was outstandingly higher than the other organs. Further, *ε*′ and *ε*″ of the lung increased temporarily on day 11. Of note, the relaxation curve of the tumor tissue at 10 GHz was similar to that of pure water, with the relaxation peak at 10 GHz (Figure 3b).

### 3.5. Where on the τ-β Diagram Is the Tumor Tissue Distributed?

**Figure 4:**
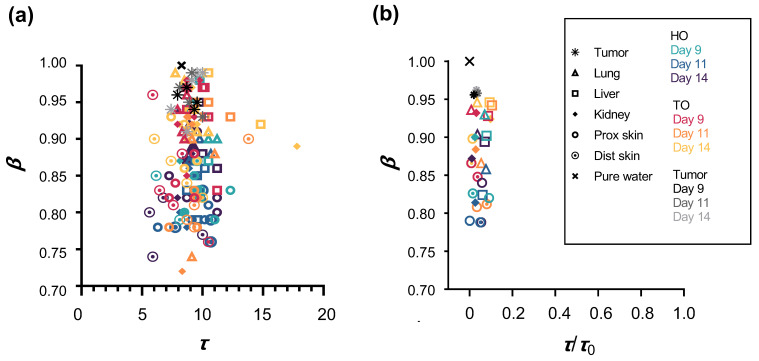
*τ*-*β* diagram of the organs and tumor tissues in healthy and tumor-bearing mice. (**a**) The distribution of plots determined by relaxation time (*τ*) and relaxation time distribution parameter (*β*). (**b**) The distribution of plots determined by the logarithm of normalized relaxation time (*τ*/*τ*_0_) and *β*.

We introduced a *τ*-*β* diagram to characterize the behavior of WSD that coexisted with macromolecules [12,13]. This plotted distribution was determined by the two parameters of *β* on the y-axis and *τ* on the x-axis. Recent interpretations have shown that the spread and fragmentation of the hydrogen bond network formed by water and surrounding molecules is related to the area plotted as a fractal dimension in the *τ*-*β* diagram. At a similar relaxation time (*τ*), the upper area of higher *β* value indicates the field of more spread and lesser fragmentation of the hydrogen bond network as a higher fractal dimension; inversely, the lower area shows the field of lesser spread and more fragmentation as a lower fractal dimension. As shown in Figure 4a, among the HO, TO, and tumor groups, most *τ* values were distributed over a narrow range of 5 to 15 ps, while the values of *β* were scattered over a relatively wider range between 0.7 and 1 in *β*. Moreover, in the logarithm of normalized relaxation time (*τ*/*τ*_0_), most *τ* values were distributed over a narrow range of 0 to 0.2, while *β* was scattered over a wider range between 0.75 and approximately 0.95 in *β* (Figure 4b).

In the *τ*-*β* diagram, the distribution plot of the tumor tissue in *β* was approximately 0.95 (close to 1 of pure water), which is higher than that of the HO and TO, indicating that tumor tissue is distributed in a higher fractal dimension with a more extensively spreading hydrogen bond network and lesser fragmentation of water molecules compared to TO and HO [12,13]. Such WSD of tumor tissue at a higher fractal dimension in the *τ*-*β* diagram is considered to correlate with finely distributed ‘free’ water.

### 3.6. Does Tumor Tissue Show Different Characteristics for Δε and β Than HO and TO?

**Figure 5:**
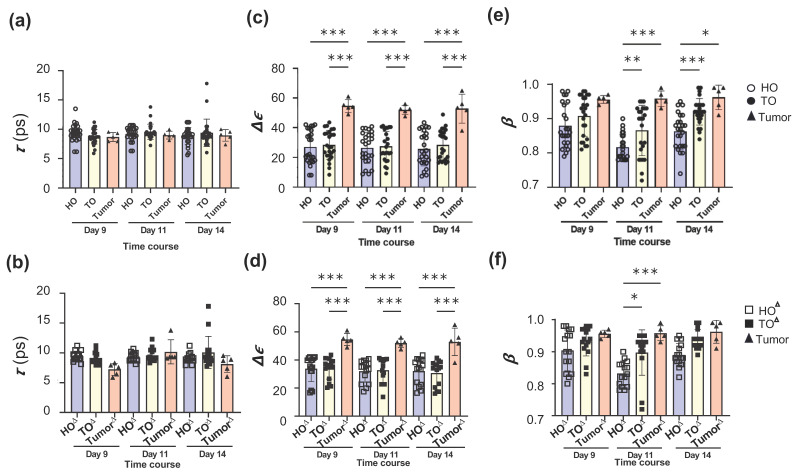
The relaxation parameters in TDR measurement in healthy and tumor-bearing mice. The comparison of each parameter among HO, TO, and tumor tissues with or without both skins. HO and TO (**a**,**c**,**e**) with both skins, and HO^∆^ and TO^∆^ (**b**,**d**,**f**) without both skins. (**a**,**b**) Relaxation time (*τ*). (**c**,**d**) Relaxation strength (∆*ε*) (**e**,**f**) Relaxation time distribution parameter (*β*). The graphs are presented as mean ± SD. n = 25 in HO or TO (**a**,**c**,**e**) and n = 15 in HO^∆^ or TO^∆^ (**b**,**d**,**f**). ^∗^ *p* < 0.05, ^∗∗∗^ *p* < 0.01, ^∗∗∗^ *p* < 0.001.

Regarding the results of Figure 3 and Figure 4, the values in HO, TO, and tumor tissue at *τ*, ∆*ε* and *β* were presented with the respective bar graphs for the whole organs (Figure 5a,c,e) and those without the prox and dist skins (Figure 5b,d,f).

At *τ*, there was no difference between the HO and TO with or without the skins and the tumor tissue (Figure 5a,b, Table S3). The respective HO and TO did not show differences (Figure S6, Table S7). However, at ∆*ε*, the tumor tissue exhibited significantly higher values than the HO and TO with or without the skins over time (Figure 5c,d, Table S4), albeit the HO and TO did not show differences (Figure S7, Table S8). Moreover, at *β*, the tumor tissue exhibited higher values than HO and TO with the skins on days 11 and 14 (Figure 5e, Table S5). When the skins were excluded, the tumor tissue exhibited significantly higher values than HO and TO only at day 11 (Figure 5f). A comparison between HO and TO showed that the *β* values of TO with or without the skins were higher than those of HO over time (Figure 5e,f, Table S9) and were significantly higher on days 11 and 14 for the whole organs and day 11 for the organs without the skins. Overall, the solid viscera, especially the liver and kidney, tended to show higher values of *β* over time, although not significantly (Figure S8). Moreover, the *β* values in the skins and lungs in TO tended to increase compared to those in HO at day 14, and significantly increased for the dist skin in TO compared to HO. Thus, the ∆*ε* and *β* values of tumor tissue increased compared to those of HO and TO, and the *β* values of TO tended to increase, to some extent, compared to those of HO.

## 4. Discussion

In this study, we revealed ex vivo WSD in tumor-bearing mice deviating from that of healthy mice using dielectric spectroscopy. The ∆*ε* value in tumor tissue was explicitly higher than that of TO and HO as comprehensive viscera (lung, liver, kidney) with or without skins; there was no difference between TO and HO. On the other hand, the *β* values in TO as well as tumor tissue tended to generally increase compared to that of HO: significantly so in TO with skins at the later period. Of interest, these findings were already recognized before macroscopic tumor growth.

### 4.1. Tumor Growth and Water Content in Tumor-Bearing and Healthy Mice

As previously reported by Penet M.F. et al. [2], the water content (%) of human pancreatic ductal adenocarcinoma xenografted in SCID mice is >80% higher than that of the healthy pancreas and other organs. The water content of mouse tumor tissues in our study corresponds to the data in the literature. Considering the water content in the TO and HO, it is understandable that WSD in the prox skin close to tumor tissue shows a similar peculiarity to that of tumor tissue, as the skin is positionally most susceptible to the tumor. Furthermore, only the water content of the prox skin in tumor-bearing mice was significantly higher than that of the orthotopic skin in healthy mice every time. To our knowledge, such investigations of water content between tumor tissues and the other organs in tumor-bearing human or mouse hosts are lacking.

### 4.2. Aberrant Dielectric Ex Vivo WSD of Tumor-Bearing Mice Versus Healthy Mice

Dielectric spectroscopy measurements revealed more outstanding differences in two parameters of WSD between tumor-bearing and healthy mice: ∆*ε* and *β* (Figure 3, Figure 4, Figure 5 and Figures S6–S8).

As previously reported, in human breast cancer cell lines, dielectric measurements at frequencies from a few GHz to over 10 GHz disclose notably higher values in dielectric constant (*ε*′), dielectric loss (*ε*″), and conductivity ($\sigma_{\mathrm{DC}}$) compared to normal mammary cell lines without culture media [26]. In human samples from patients with colon [15] and hepatocellular [16] carcinoma, dielectric measurements exhibited unique patterns with a higher dielectric constant at similar frequencies.

The complex dielectric constant of colorectal mucosal lesions increases as they progress from normal to benign to malignant [15]. Moreover, the complex dielectric constant transition does not correlate with lesion shape or size. Similarly, in the present study, the ∆*ε* value of tumor tissues was outstandingly higher than that of HO and TO with or without skins to the same degree over time without any association with tumor growth. In this way, ‘microscopic’ ∆*ε* reflecting water molecular density was considered to capture measurements more sensitively than ‘macroscopic’ water content. Further, the *β* value also tended to increase toward 1 in tumor tissues compared to HO and TO. Of note, the *β* value of TO tended to be higher than that of HO every time, which became more prominent in organs with skin included.

The consistently higher ∆*ε* value in tumor tissues through the period is considered to probably reflect the intracellular hydration of unlimitedly proliferative tumor cells, due to the higher ∆*ε* at the proliferative S and G_2_ phases in cell cycle [10].

Moreover, such tumors with the intensive movement of water molecules may more or less affect the movement of water molecules not only in the surrounding skins, but also even the remote organs (viscera). There was no difference in ∆*ε* value between TO and HO with or without the skins. However, skin-including TO and tumor tissues disclosed higher *β* values compared to skin-including HO later in the period; the remote viscera of TO and tumor tissue tended to show higher *β* values compared to those of HO. The findings suggest that WSD in tumors as well as the viscera is prone to shift to the higher fractal dimension in the *τ*-*β* diagram, indicating easily spreading of the hydrogen bond network with less fragmentation. Furthermore, considering *β* and ∆*ε* as qualitative and quantitative relaxation parameters expressing WSD of water molecules, respectively, the former might be more likely to be affected in a tumor-bearing living body. Collectively, it is noteworthy that the dynamic changes in water molecules in tumor-bearing mice had already occurred during early tumor engraftment rather than as the tumor grew. Such aberrant WSD in tumor tissues and distant main organs like the liver and kidney in tumor-bearing mice suggests that a malignant tumor is not a localized disease but a systemic disease, regardless of tumor growth.

### 4.3. The Pathophysiological Mechanism of Aberrant Dielectric Ex Vivo WSD in Tumor-Bearing Mice

Water structure analysis using dielectric spectroscopy revealed a higher ∆*ε* value in surgically resected colon [14,15], liver [16] or brain [17] tumors, presumably, in part, reflecting the higher cellularity in tumor tissues, as with aggregated breast epithelial cells in 3D culture [27]. The remarkably high ∆*ε* value of tumor tissue in the present study may reflect the crowded B16 melanoma cells and the interstitial cells like cancer-associated fibroblasts in the tumor microenvironment [28]. Alternatively, malignant tumors generally induce premature angiogenesis in the tumor microenvironment to supply nutrients to the tumor and surrounding tissues, resulting in increased vascular permeability and abnormal microcirculation [29,30]. This is anticipated to induce ‘hyperhydration’, leading to enriched water-molecule density. As expected, the proximal skin in the mice exhibited similar dielectric features to tumor tissues. In our previous study of the sciatic nerve crush injury model [18], the reduction in dielectric impedance in the proximal skin is similar to that in crush injury nerves caused by inflammation, edema, and vascular hyperpermeability. In the present study, the similar dielectric features in the proximal skin and tumors may be caused by the hyperpermeability and inflammation in the tumor tissues, including the proximal skin. Therefore, the increased cell density in tumor tissues with hyperpermeability in the tumor microenvironment may cause the aberrant WSD of the tumor tissues. Moreover, various inflammatory and angiogenic cytokines, e.g., TNF-α, interferon-γ and VEGF etc. [30,31,32], secreted from tumor tissue, flow into the circulation, suggesting an effect on the WSD of systematic viscera or vasculartures in tumor-bearing bodies. Such a pathological cytokine environment might also lead to the aberrant WSD in a tumor-bearing body.

### 4.4. Different Dielectric Ex Vivo WSD of Tumor-Bearing and Septic Mice

We previously classified WSD in water coexisting with some molecules into two groups: a ‘solution system’ and a ‘dispersion system’ [12,13]. WSD can be represented on a two-dimensional plot defined by *β* on the y-axis and the logarithm of *τ*/*τ*_0_ on the x-axis, which is called a ‘*τ*-*β* diagram’. The ‘Solution system’ is distributed over the scattered *τ*/*τ*_0_ with the higher *β* and the ‘dispersion system’ over the lower *τ*/*τ*_0_ with the scattered *β*. Glucose or collagen is categorized as a ‘solution system’, and gelatin or glass eggs as a ‘dispersion system’.

We showed ex vivo mouse organs categorized in the ‘dispersion system’ in a *τ*-*β* diagram [12,13]. Moreover, the WSD of the organs in septic mice shifted to that of elongated τ and lower *β* compared to those of healthy mice. In the present study, TO and HO belonged to the ‘dispersion system’, similar to the septic mice. However, the WSD of tumor tissues and TO shifted to the position with the higher *β* and without elongation of *τ*/*τ*_0_, unlike that in septic mice organs. WSD in tumor tissues is particularly conspicuous and may be classified into a dual system: the ‘dispersion system’ and ‘solution system’. This indicates that septic and tumor-bearing living bodies have the respective characteristic WSDs, suggesting that the difference depends on individual diseases.

### 4.5. Study Limitations and Future Experiments

The study measured WSD ex vivo post-sacrifice and may not accurately capture in vivo WSD in living mice. A methodology to evaluate the WSD of tumors and organs of live mice is expected to be developed. Furthermore, the following intriguing issues need to be elucidated in future experiments in conjunction with biochemical analyses, including histology: (1) the pathophysiological impact of WSD abnormalities in tumor-bearing mice, (2) the cause of the WSD abnormality in tumor tissue and organs in tumor-bearing mice; whether the aberrant WSD is due to tumor growth or the latter due to the former, (3) the feature of WSD with tumor growth at the extended observation time, or with any antitumor treatment, (4) exploring WSD in metastatic models.

## 5. Conclusions

Dielectric spectroscopy using TDR revealed the aberrant WSD of the tumor and the internal organs in a primary tumor model even before macroscopically noticeable tumor growth. Thus, this material–physical analysis may provide a potential methodology to provide novel pathophysiological information about malignant tumors.

## Data Availability

The data presented in this study are available on reasonable request from the corresponding author.

## Supplementary Materials

The following supporting information can be downloaded at: https://www.mdpi.com/article/10.3390/biology12091250/s1, Figure S1: The frequency dependency of complex permittivity presented by the Debye formula; Figure S2: The function given by the curve fitting analysis of dielectric relaxation measurement; Figure S3: Curve fitting analysis of tumor tissues; Figure S4: The water contents in each organ of healthy and tumor-bearing mice; Figure S5: The consistency of the dielectric constant and conductivity of the measured values to online human database in each organ; Figure S6: The relaxation time in each organ of healthy and tumor-bearing mice; Figure S7: The relaxation strength in each organ of healthy and tumor-bearing mice. Figure S8. The relaxation time distribution parameter in each organ of healthy and tumor-bearing mice; Table S1: Tumor sizes and weights over time; Table S2: Water contents of tumor tissues and the organs in HO and TO; Table S3: Relaxation time (*τ*) of tumor tissues and the organs in HO and TO; Table S4: Relaxation strength (∆*ε*) of tumor tissues and the organs in HO and TO; Table S5: Relaxation time distribution parameter (*β*) of tumor tissues and the organs in HO and TO; Table S6: Water contents in each organ of HO and TO; Table S7: Relaxation time (*τ*) in each organ of HO and TO; Table S8: Relaxation strength (∆*ε*) in each organ of HO and TO; Table S9: Relaxation time distribution parameter (*β*) in each organ of HO and TO.
