# Supplementary material for: Aberrant Water Structure Dynamics in B16 Melanoma-Bearing Mice by Time Domain Refractometry Analysis
Source: Biology (Basel). 2023 Sep 18;12(9):1250. doi: 10.3390/biology12091250 (PMC10525127; doi:10.3390/biology12091250)
Supplement: Supplementary file 1 [file biology-12-01250-s001.zip › 20230726 Supplemental Materials.pdf]

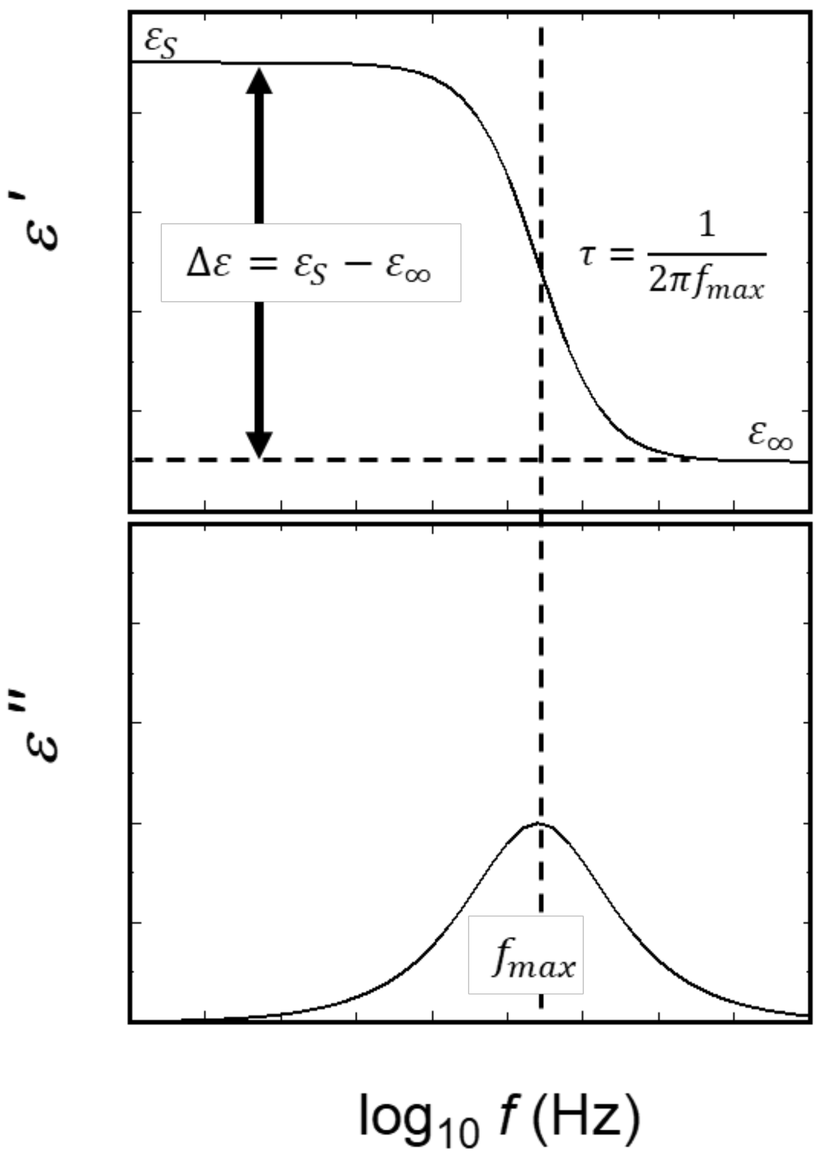

Figure S1. The frequency dependency of complex permittivity presented by the Debye formula. Relaxation time (τ) is given by $\tau = 1/2\pi f_{max}$. f_{max} indicates specific peak frequency.

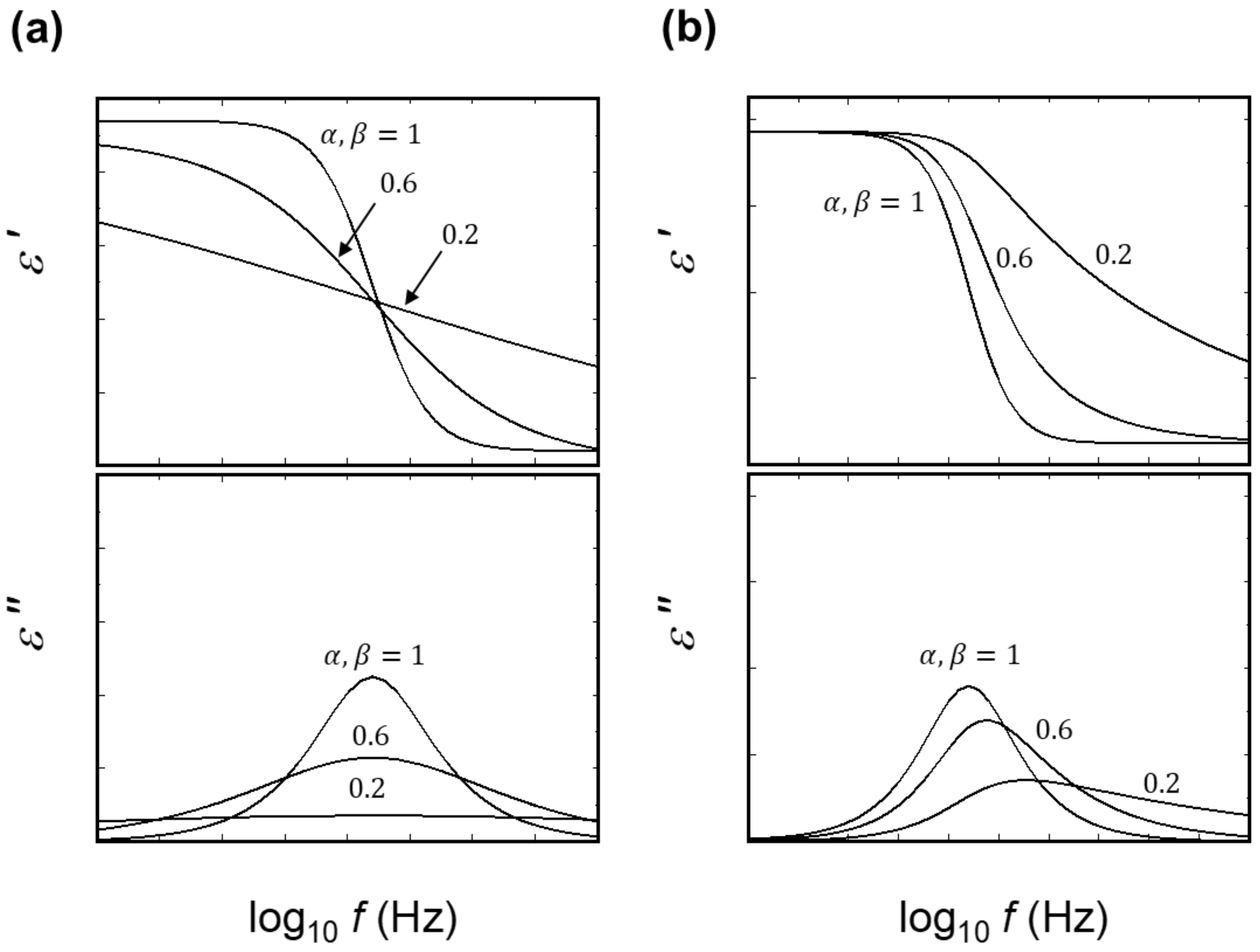

Figure S2. The function given by the curve fitting analysis of dielectric relaxation measurement.
(a) The frequency dependency of complex permittivity presented by the Cole-Cole formula. The distribution of relaxation time distribution parameter (β) shows a symmetrical distribution, ranging from >0 to ≤1 . The closer to 1, the more uniform the structure. (b) The frequency dependency of complex permittivity presented by the Davidson-Cole formula. The distribution of relaxation time dispersion is presented by asymmetrical distribution (α) and symmetrical distribution (β): both distributions ranged between >0 and ≤1. The closer to 1 at β, the more uniform the structure.

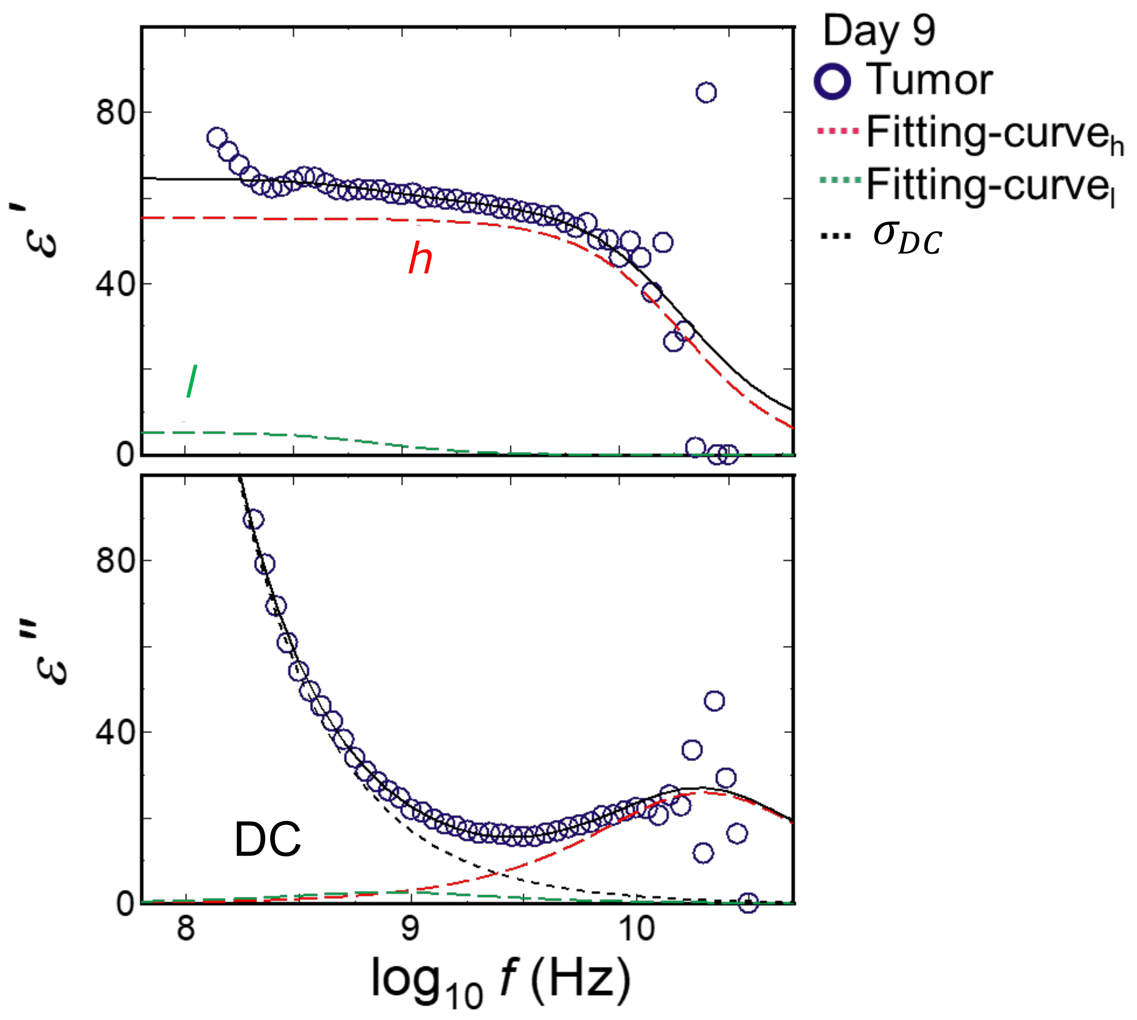

Figure S3. Curve fitting analysis of tumor tissues.

The relaxation curve was divided into the lower and higher frequency processes (*l* and *h*) on tumor tissues on day 9. The relaxation parameters, τ, β, and $\Delta\varepsilon$ for *l*- and *h*-processes were determined by curve fitting analysis using two Cole-Cole formulas with a contribution of the DC conductivity term. Then, DC indicates direct electrical conduction of *in vivo* macromolecules and ions derived from living organisms. The analysis was performed in the frequency range from 100 MHz to 30 GHz.

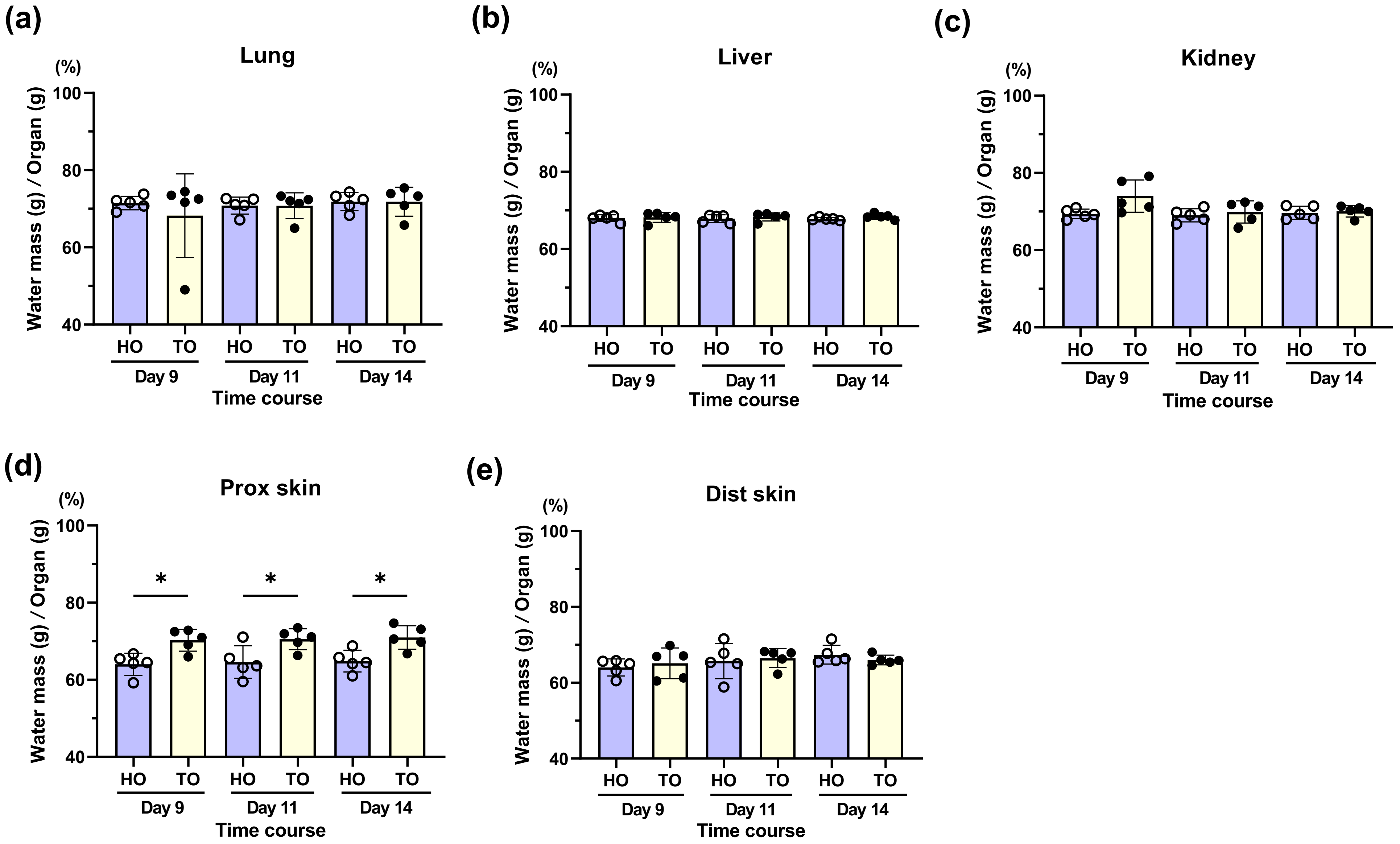

Figure S4. The water contents in each organ of healthy and tumor-bearing mice.
The graphs are presented as mean ± SD. n=5 in HO or TO. *$p < 0.05$.

(a)

Dielectric constant (ε’) at around 1GHz						
		Kidney	Liver	Lung	Dist skin	Prox skin
Online database		57.9	46.4	21.8	40.9	
Healthy organ	day 9	40.49	46.52	24.24	22.81	26.01
	day 11	39.55	36.6	24.52	35.74	26.85
	day 14	39.79	46.18	28.33	25.67	22.18
Tumor organ	day 9	43.82	46.27	33.21	25.21	33.02
	day 11	41.35	47.17	36.5	23.19	24.34
	day 14	42.18	45.94	21.98	29.54	24.88

(b)

Conductivity (σDC) at around 1GHz						
		Kidney	Liver	Lung	Dist skin	Prox skin
Online database		1.450	0.897	0.474	0.900	
Healthy organ	day 9	0.730	0.766	0.461	0.341	0.434
	day 11	0.827	0.750	0.534	0.748	0.509
	day 14	0.780	0.763	0.604	0.480	0.395
Tumor organ	day 9	0.892	0.834	0.715	0.439	0.627
	day 11	0.799	0.813	0.703	0.389	0.422
	day 14	0.842	0.786	0.428	0.565	0.481

(c)

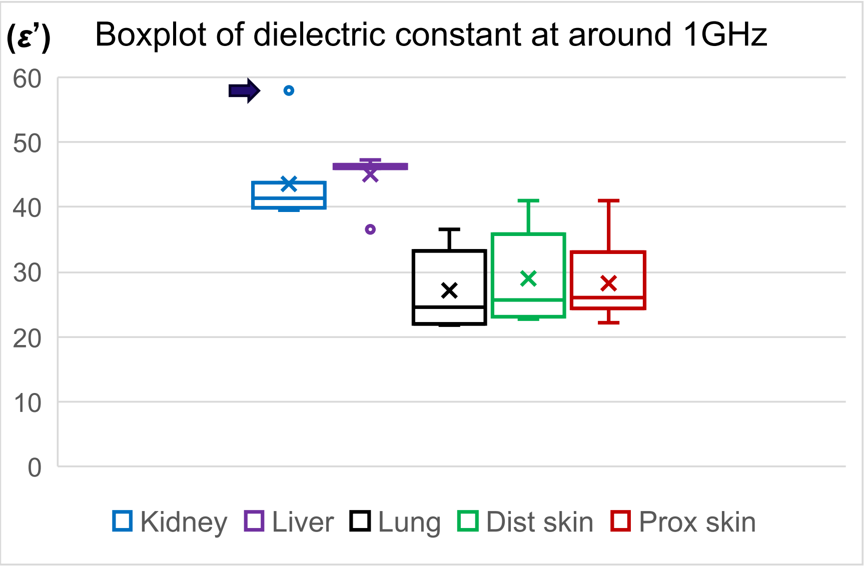

(d)

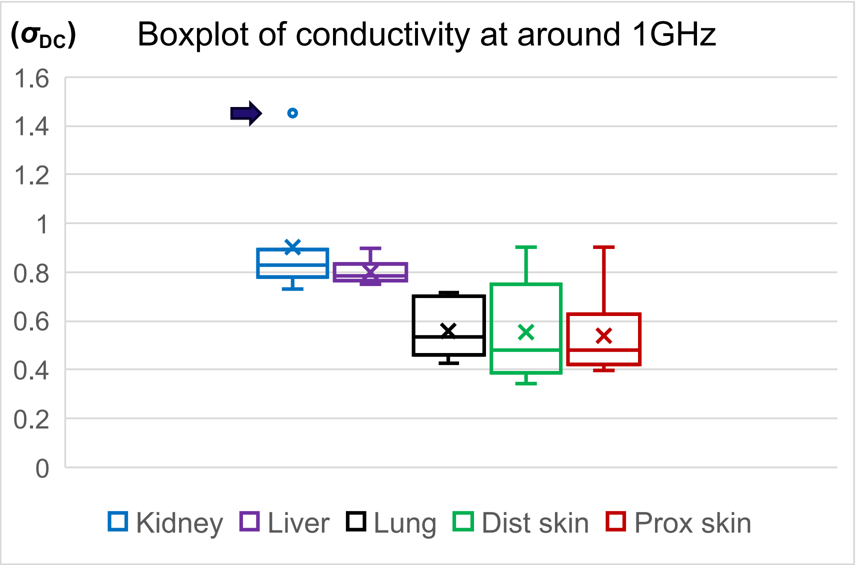

Figure S5. The consistency of the dielectric constant and conductivity of the measured values to online human database in each organ. The top tables indicate the values of dielectric constant (a) and conductivity (b) at around 1GHz in each organ of the present experiment and online database. The representative values in each of dielectric constant and conductivity in the present experiment are compared to those of online database. The bottom graphs (c. d) indicate the boxplots from the tables of dielectric constant (a) and conductivity (b) respectively. The boxplot graph of each organ is formed of the whole values of the present experiment and online database. In (c) and (d), the arrowheads indicate the outliners of online database.

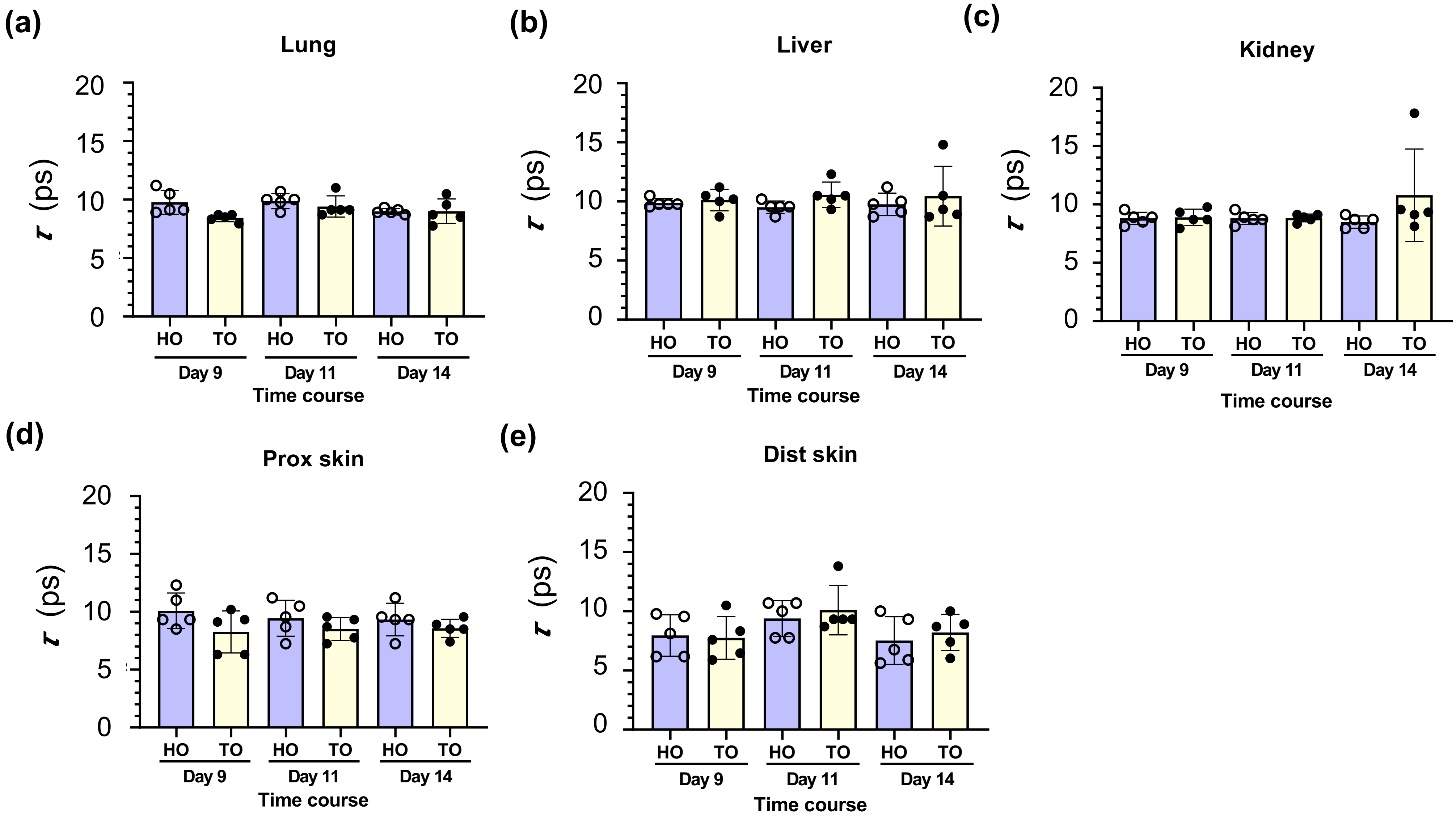

Figure S6. The relaxation time (τ) in each organ of healthy and tumor-bearing mice. The graphs are presented as mean ± SD. n=5 in HO or TO.

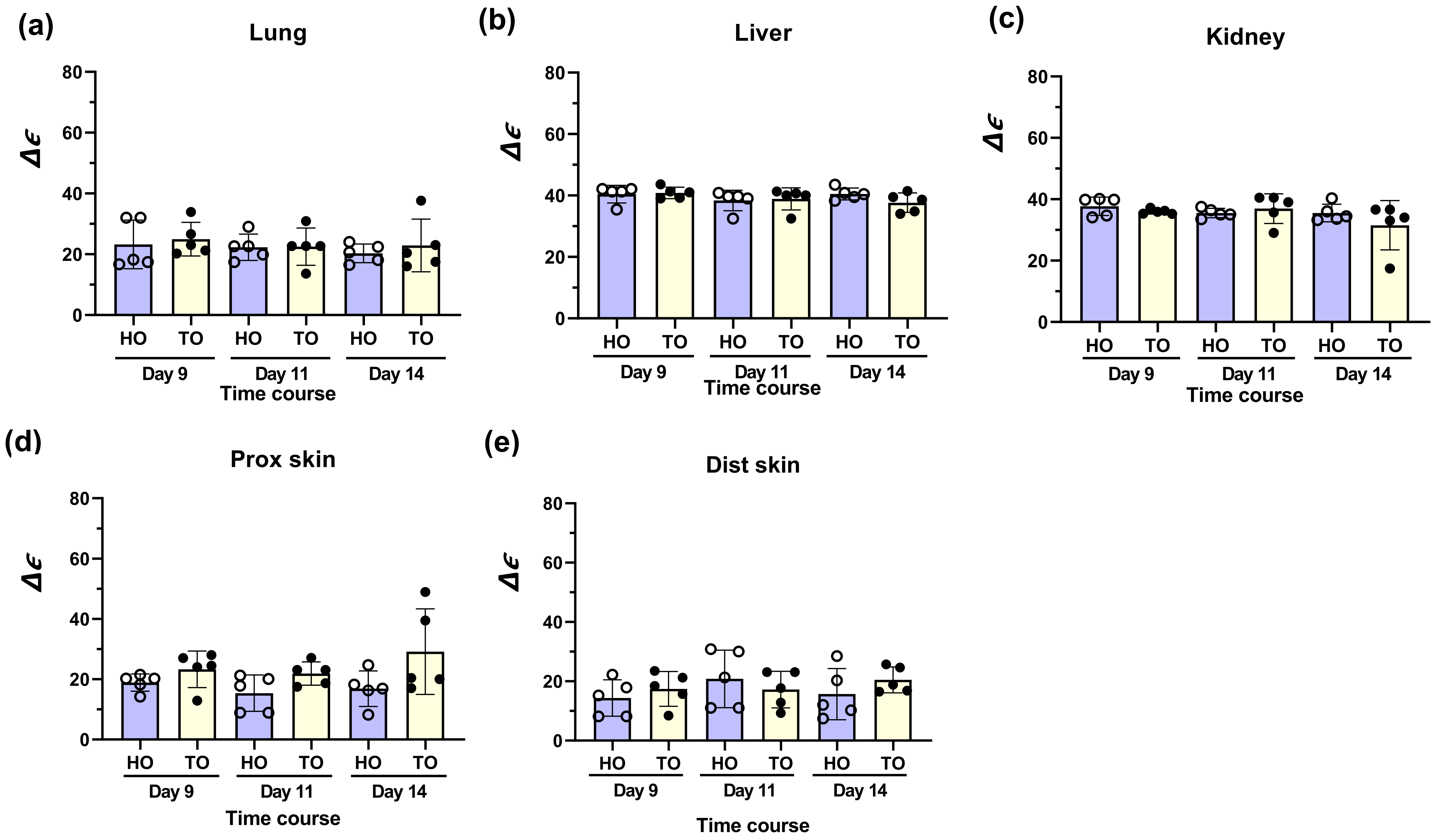

Figure S7. The relaxation strength (*Δϵ*) in each organ of healthy and tumor-bearing mice. The graphs are presented as mean±SD. n=5 in HO or TO.

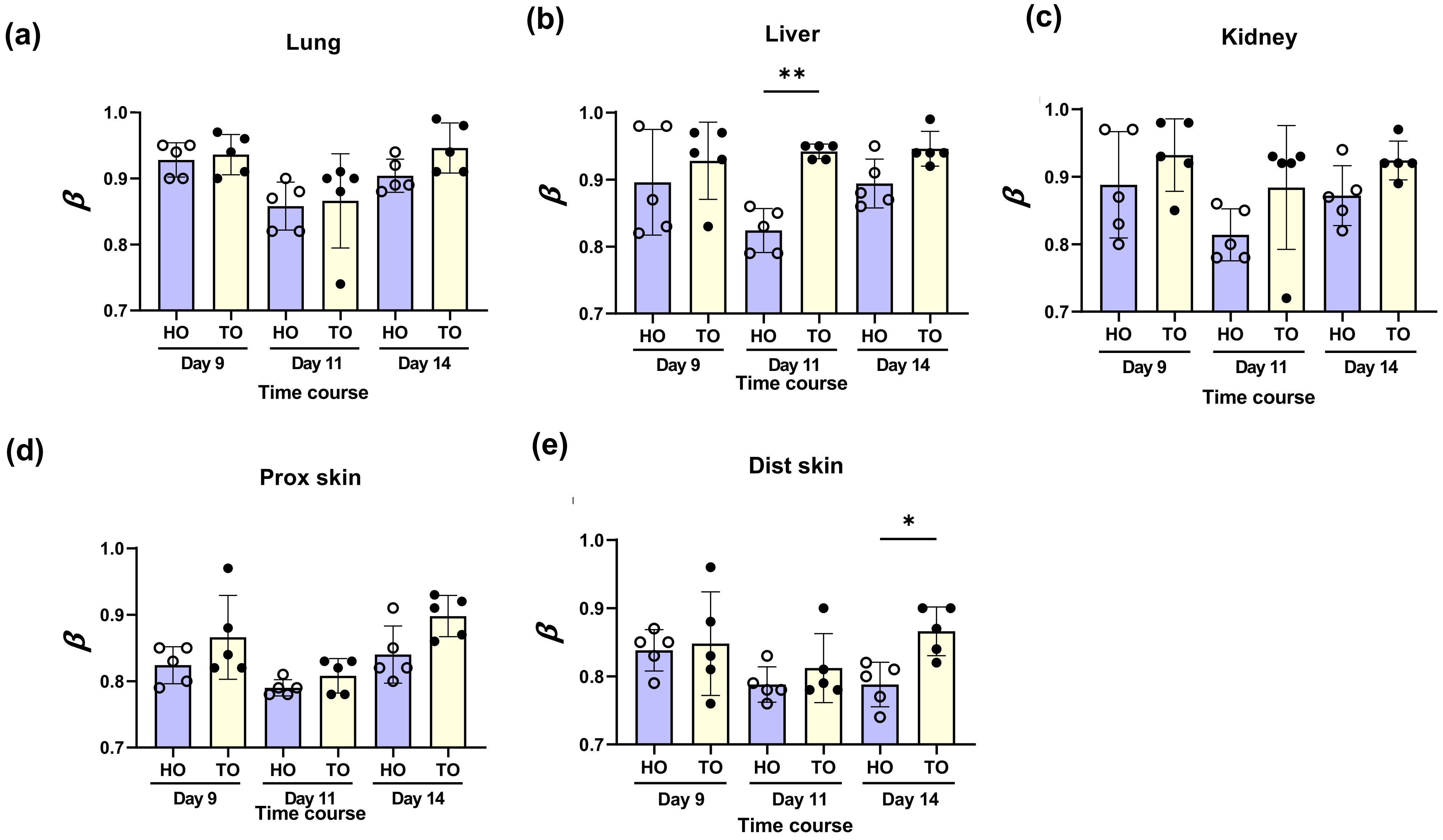

Figure S8. The relaxation time distribution parameter (*β*) in each organ of healthy and tumor-bearing mice. The graphs are presented as mean ± SD. n=5 in HO or TO. $*p < 0.05$, $**p < 0.01$.

Table S1. Tumor sizes and weights during the observation period.

Tumor size (mm^3)			Tumor weight (g)		
Day 9	Day 11	Day 14	Day 9	Day 11	Day 14
301.8±123.6	314.1±116.5	581.5±247.6	0.138±0.090	0.213±0.229	0.274±0.264

Table S2. Water contents of tumor tissues and the organs in HO and TO.

	Whole organs (%)		The other organs than skins (%)		Tumor (%)
	HO	TO	HO	TO	
Day 9	67.4 ± 3.5	69.2 ± 6.0	69.6 ±1.9	70.1 ± 6.8	72.8 ± 6.6
Day 11	67.6 ± 3.6	67.9 ± 7.5	69.2 ± 2.0	67.6 ± 9.5	75.3 ± 3.7
Day 14	68.3 ± 3.1	69.5 ±3 .0	69.8 ± 2.3	70.1 ± 2.6	75.2 ± 4.3

Table S3. Relaxation time (τ) of tumor tissues and the organs in HO and TO.

	Whole organs (ps)		The other organs than skins (ps)		Tumor (ps)
	HO	TO	HO	TO	
Day 9	9.39 ± 1.51	8.74 ± 1.25	9.48 ± 1.02	9.15 ± 0.32	8.69 ± 0.75
Day 11	9.22 ± 1.23	9.58 ± 1.30	9.40 ± 0.64	9.60 ± 0.89	9.02 ± 0.67
Day 14	8.87 ± 1.43	9.41 ± 2.33	9.08 ± 0.24	10.08 ± 1.05	8.96 ± 0.99

Table S4. Relaxation strength ($\Delta\varepsilon$) of tumor tissues and the organs in HO and TO.

	Whole organs		The other organs than skins		Tumor
	HO	TO	HO	TO	
Day 9	27.0 ± 11.3	28.5 ± 9.7	33.7 ± 9.1	33.9 ± 7.6	54.6 ± 4.3
Day 11	26.5 ± 10.5	27.5 ± 10.0	32.0 ± 7.8	32.8 ± 8.9	52.0 ± 3.0
Day 14	25.8 ± 11.4	28.3 ± 10.1	32.1 ± 9.2	30.7 ± 9.1	52.9 ± 9.7

Table S5. Relaxation time distribution parameter (β) of tumor tissues and the organs in HO and TO.

	Whole organs		The other organs than skins		Tumor
	HO	TO	HO	TO	
Day 9	0.876 ± 0.063	0.902 ± 0.065	0.911 ± 0.056	0.932 ± 0.045	0.956 ± 0.011
Day 11	0.815 ± 0.038	0.862 ± 0.073	0.832 ± 0.039	0.897 ± 0.071	0.958 ± 0.023
Day 14	0.860 ± 0.055	0.916 ± 0.043	0.890 ± 0.036	0.939 ± 0.031	0.962 ± 0.036

Table S6. Water contents in each organ of HO and TO.

	HO (%)					TO (%)				
	Lung	Liver	Kidney	Dist skin	Prox skin	Lung	Liver	Kidney	Dist skin	Prox skin
Day 9	71.5 ± 1.7	68.0 ± 0.9	69.4 ± 1.2	64.0 ± 2.2	64.0 ± 2.9	68.2 ± 10.8	68.2 ± 1.3	74.0 ± 4.2	65.1 ± 4.1	70.3 ± 2.8
Day 11	70.8 ± 2.2	67.9 ± 1.0	69.0 ± 1.7	65.7 ± 4.7	64.6 ± 4.2	70.8 ± 3.3	68.3 ± 1.0	63.6 ± 16.5	66.5 ± 2.5	70.5 ± 2.7
Day 14	71.8 ± 2.3	67.8 ± 0.4	69.7 ± 1.7	67.4 ± 2.5	64.8 ± 2.8	71.8 ± 3.8	68.4 ± 0.7	70.1 ± 1.5	66.0 ± 1.3	71.0 ± 3.1

Table S7. Relaxation time (τ) in each organ of HO and TO.

	HO (ps)					TO (ps)				
	Lung	Liver	Kidney	Dist skin	Prox skin	Lung	Liver	Kidney	Dist skin	Prox skin
Day 9	9.77 ± 1.02	9.92 ± 0.33	8.76 ± 0.56	8.19 ± 2.07	10.33 ± 1.98	8.44 ± 0.32	10.12 ± 0.91	8.89 ± 0.70	7.75 ± 1.81	8.48 ± 0.94
Day 11	9.88 ± 0.64	9.52 ± 0.54	8.80 ± 0.51	9.39 ± 1.51	8.49 ± 2.06	9.41 ± 0.89	10.56 ± 1.08	8.84 ± 0.34	10.10 ± 2.09	9.00 ± 0.99
Day 14	9.00 ± 0.24	9.76 ± 0.96	8.49 ± 0.52	7.52 ± 2.02	9.58 ± 1.70	9.01 ± 1.05	10.45 ± 2.53	10.79 ± 3.96	8.21 ± 1.53	8.58 ± 0.78

Table S8. Relaxation strength (Δε) in each organ of HO and TO.

	HO					TO				
	Lung	Liver	Kidney	Dist skin	Prox skin	Lung	Liver	Kidney	Dist skin	Prox skin
Day 9	23.2 ± 8.0	40.4 ± 2.8	37.3 ± 2.8	14.7 ± 6.3	19.3 ± 2.9	25.0 ± 5.6	40.8 ± 1.9	36.0 ± 0.8	17.4 ± 5.8	23.3 ± 6.0
Day 11	22.3 ± 4.3	38.3 ± 3.3	35.5 ± 1.5	20.8 ± 9.7	15.4 ± 6.0	22.5 ± 6.1	38.9 ± 3.6	36.9 ± 4.9	17.2 ± 6.2	21.9 ± 3.9
Day 14	20.3 ± 3.1	40.5 ± 1.9	35.5 ± 2.9	15.7 ± 8.6	16.9 ± 5.9	22.9 ± 8.6	37.7 ± 3.2	31.5 ± 8.1	20.5 ± 4.4	29.2 ± 14.2

Table S9. Relaxation time distribution parameter (β) in each organ of HO and TO.

	HO					TO				
	Lung	Liver	Kidney	Dist skin	Prox skin	Lung	Liver	Kidney	Dist skin	Prox skin
Day 9	0.930 ± 0.023	0.902 ± 0.074	0.900 ± 0.066	0.826 ± 0.026	0.820 ± 0.028	0.936 ± 0.030	0.928 ± 0.058	0.932 ± 0.054	0.848 ± 0.076	0.866 ± 0.063
Day 11	0.858 ± 0.036	0.824 ± 0.033	0.814 ± 0.038	0.788 ± 0.026	0.790 ± 0.012	0.866 ± 0.071	0.942 ± 0.011	0.884 ± 0.092	0.812 ± 0.051	0.808 ± 0.026
Day 14	0.904 ± 0.025	0.894 ± 0.036	0.872 ± 0.044	0.788 ± 0.033	0.840 ± 0.043	0.946 ± 0.038	0.946 ± 0.026	0.924 ± 0.029	0.866 ± 0.036	0.898 ± 0.031
